# Human Pluripotent Stem Cell-Derived Mesenchymal Stem Cells Prevent Allergic Airway Inflammation in Mice

**DOI:** 10.1002/stem.1241

**Published:** 2012-09-17

**Authors:** Yue-Qi Sun, Meng-Xia Deng, Jia He, Qing-Xiang Zeng, Weiping Wen, David SH Wong, Hung-Fat Tse, Geng Xu, Qizhou Lian, Jianbo Shi, Qing-Ling Fu

**Affiliations:** a Otorhinolaryngology Hospital, The First Affiliated Hospital, Sun Yat-sen UniversityGuangzhou, Guangdong, People's Republic of China; b Cardiology Division, Department of Medicine and Research Centre of Heart, Brain, Hormone, and Healthy Aging, The University of Hong KongHong Kong; c Department of Ophthalmology, The University of Hong KongHong Kong

**Keywords:** Induced pluripotent stem cells, Mesenchymal stem cells, Allergy, Immunomodulation

## Abstract

We previously found that mesenchymal stem cells (MSCs) derived from human-induced pluripotent stem cells (iPSCs) exerted immunomodulatory effects on Th2-mediated allergic rhinitis in vitro. However, their contribution to the asthma and allergic rhinitis in animal models remains unclear. In this study, we developed a mouse model of ovalbumin (OVA)-induced allergic inflammation in both the upper and lower airways and evaluated the effects of the systemic administration of human iPSC-MSCs and bone marrow-derived MSCs (BM-MSCs) on allergic inflammation. Our results showed that treatments with both the iPSC-MSCs and BM-MSCs before the challenge phase protected the animals from the majority of allergy-specific pathological changes. This protection included an inhibition of inflammatory cell infiltration and mucus production in the lung, a reduction in eosinophil infiltration in the nose, and a decrease in inflammatory cell infiltration in both the bronchoalveolar and nasal lavage fluids. In addition, treatment with iPSC-MSCs or BM-MSCs before the challenge phase resulted in reduced serum levels of Th2 immunoglobulins (e.g., IgE) and decreased levels of Th2 cytokines including interleukin (IL)-4, IL-5, or IL-13 in the bronchoalveolar and/or nasal lavage fluids. Similar therapeutic effects were observed when the animals were pretreated with human iPSC-MSCs before the sensitization phase. These data suggest that iPSC-MSCs may be used as an alternative strategy to adult MSCs in the treatment of asthma and allergic rhinitis. Stem Cells 2012;30:2692–2699

## INTRODUCTION

Mesenchymal stem cells (MSCs) are multipotent cells that are capable of differentiating into three types of mesenchymal cells: adipocytes, osteoblasts, and chondrocytes [1]. Increasing evidence in animal studies and in preliminary clinical trials has demonstrated that MSCs not only possess multipotent differentiation potential but also exhibit strong immunomodulation potential via their interaction with T lymphocytes, B lymphocytes, natural killer (NK) cells, and dendritic cells (DC) [2–4]. Adult bone marrow-derived MSCs (BM-MSCs) were initially reported and have been the main source for the isolation of MSCs. However, there are several potential limitations of using adult MSCs, including their limited capacity to proliferate, the significant variability in cell quality derived from different donors and a rapid loss of their differentiation potential [5–7]. In addition, aging and aging-related disorders significantly impair the survival and differentiation potential of BM-MSCs, thus limiting their therapeutic efficacy [8, 9].

We recently succeeded in inducing MSCs from human induced pluripotent stem cell (iPSCs) [10]. iPSC-MSCs not only express well-known adult BM-MSC markers and display the potential for adipogenesis, osteogenesis, and chondrogenesis but also displayed a higher capacity for both proliferation and telomerase activity. In addition, iPSC-MSCs were superior in the repair of tissue ischemia via paracrine and transdifferentiation mechanisms compared with their adult BM-MSC counterparts [10]. iPSC-MSCs have been demonstrated to display significant inhibition of NK cell proliferation and cytolytic function [11]. However, the anti-inflammatory or immunomodulatory properties of iPSC-MSCs have not been defined in vivo.

Asthma and allergic rhinitis (AR) are chronic, reversible allergic airway diseases that have become a significant global public health concern [12]. According to the Global Initiative for Asthma, approximately 300 million people suffer from asthma, resulting in substantial medical and financial burdens [13, 14]. An increasing body of evidence indicates that the upper and lower airways share common inflammatory mechanisms [15], including eosinophilic inflammation in the subepithelial mucosa and Th2 skewing of the immune response [16]. Adult MSCs have been demonstrated to suppress allergic airway inflammatory diseases, including asthma [17–20] and AR [21, 22], in animal models. We previously found that similar to BM-MSCs, iPSC-MSCs significantly inhibited the levels of Th2 cytokines including interleukin (IL)-4, IL-5, and IL-13 and promoted regulatory T cell responses after coculture with peripheral blood mononuclear cells of AR patients [23]. The potential role of iPSC-MSCs in attenuating allergic airway inflammation requires further investigation in animal models. In this study, we developed a mouse model of allergic inflammation in both the upper and lower airways, and the effects of the systemic administration of human iPSC-MSCs compared with BM-MSCs on allergy-specific pathology and Th2 cytokines were evaluated.

## MATERIALS AND METHODS

### Animals

Female BALB/c mice (4–6 weeks of age) were purchased from Experimental Animal Center, Sun Yat-sen University (Guangzhou, People's Republic of China) and housed under specific pathogen-free conditions. All procedures were performed according to protocols approved by the Institutional Animal Care and Use Committee, Sun Yat-sen University (No. IACUC 20110228002).

### Preparation of Human iPSC-MSCs and BM-MSCs and Flow Cytometry Analysis of Surface Marker Expression

Two clones of iPSC-MSCs, iMR90-iPSC-MSCs 10 [10] and N1-iPSC-MSCs [24], were used in this study. iMR90-iPSC-MSCs 10 were generated from iPSC-iMR90-5 (WiCell Research Institute, Madison, WI, http://www.wicell.org) [10]. The N1-iPSC-MSC clone was prepared from iPSCs reprogrammed from human fibroblast cells as shown in our previous study [24]. The iPSCs were differentiated into MSCs according to the protocol previously described [25]. Briefly, MSCs were purified by sorting for CD105+/CD24− cells and were maintained in medium containing 90% knockout Dulbecco's modified Eagle's medium (Gibco, Invitrogen Corporation, Carlsbad, CA, http://www.invitrogen.com) supplemented with 10% serum replacement medium (Gibco) and basic fibroblast growth factor (10 ng/ml, Gibco). MSC identity was verified by surface marker expression of CD24, CD34, CD31, CD44, CD73, CD29, CD105, and CD166 using phycoerythrin (PE)- or fluorescein isothiocyanate (FITC)-conjugated antibodies (BD Biosciences, San Jose, NJ, http://www.bdbiosciences.com). The iPSC-MSCs were morphologically highly similar to BM-MSCs and had similar surface antigen expression, including expression of CD44, CD49a and e, CD73, CD105, and CD166 and lack of expression of CD45, CD34, and CD133. Furthermore, the iPSC-MSCs were identified to have the capacity to differentiate into osteoblasts, adipocytes, and chondroblasts. Human BM-MSCs were purchased commercially and served as the control cells (Cambrex BioScience, Rockland, ME, http://www.cambrex.com; catalog number PT-2501). We used the MSCs at the fourth through the sixth passages for transplantation.

### Mouse Model of Allergic Airways Inflammation

**Figure 1:**
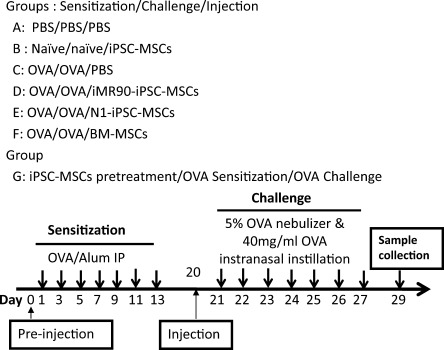
Schematic diagram showing the mouse model of allergic airway inflammation. Mice were sensitized on days 1, 3, 5, 7, 9, 11, and 13 by intraperitoneal injection of OVA with aluminum hydroxide. From days 21 to 27, the mice were challenged with aerosolized 5% OVA in a plexiglass chamber for 30 minutes and subsequently intranasally exposed to 20 μl of OVA (40 mg/ml) each day. Purified iMR90-iPSC-MSCs, N1-iPSC-MSCs, or BM-MSCs (1 × 10^6^) were injected via the tail vein on day 20. Moreover, human iMR90-iPSC-MSCs (1 × 10^6^) were preinjected via the tail vein on day 0. Samples were collected on day 29. The mice were divided into seven different groups in accordance with the different treatments of sensitization, challenge and injection or preinjection, sensitization and challenge. Abbreviations: BM-MSC, bone marrow-derived mesenchymal stem cell; iPSC-MSC, induced pluripotent stem cell-derived mesenchymal stem cell; OVA, ovalbumin; PBS, phosphate-buffered saline.

A mouse model of allergic airways inflammation was induced as previously reported with minor modification [26]. Briefly, mice were sensitized by intraperitoneal injection of 40 μg of ovalbumin (OVA, grade V, Sigma, St. Louis, MO, http://www.sigmaaldrich.com) in 2 mg of aluminum hydroxide (Sigma) in 200 μl pyrogen-free phosphate-buffered saline (PBS) on days 1, 3, 5, 7, 9, 11, and 13, as shown in Figure 1. From days 21 to 27, mice were challenged daily with aerosolized 5% OVA in a plexiglass chamber and through an air-compressing nebulizer (403A, yuyue, Danyang, Jiangsu, People's Republic of China, http://www.yuyue.com.cn) for 30 minutes. Subsequently, mice were intranasally infused with 20 μl OVA (40 mg/ml). The animals were sacrificed via cervical dislocation at day 29.

### Human iPSC-MSCs or BM-MSCs Transplantation

Two clones of human iPSC-MSCs, iMR90-iPSC-MSCs and N1-iPSC-MSCs or human BM-MSCs were suspended in sterile PBS at a density of 5 × 10^6^ cells per ml. On day 0 or 20, 0.2 ml of cells or of the sterile PBS was injected via the tail vein (Fig. 1). Mice were divided into seven groups: (A) PBS/PBS/PBS mice that were sensitized and challenged with PBS were then injected with PBS on day 20 (*n* = 5); (B) naïve/naïve/iPSC-MSCs mice that were naïve mice treated with iPSC-MSCs (*n* = 5); (C) OVA/OVA/PBS mice that were sensitized and challenged with OVA, then treated with PBS on day 20, (*n* = 5); (D) OVA/OVA/iMR90-iPSC-MSC mice that were sensitized and challenged with OVA and then treated with iMR90-iPSC-MSCs (*n* = 6); (E) OVA/OVA/N1-iPSC-MSC mice that were sensitized and challenged with OVA and then treated with N1-iPSC-MSCs on day 20 (*n* = 5); (F) OVA/OVA/BM-MSC mice that were sensitized and challenged with OVA and then treated with BM-MSCs (*n* = 4); (G) iPSC-MSC/OVA/OVA mice that were treated with iMR90-iPSC-MSCs on day 0, and then sensitized, challenged with OVA (*n* = 5).

### Evaluation of Nasal Symptoms

The frequency of sneezing and nose rubbing that occurred in the 10-minute time period after the last challenge was determined as previously reported [27]. Mice were subjected to a single-blind observation by examiners who had no knowledge of the experimental groups.

### Evaluation of the Inflammatory Cells and Cytokines in the Nasal and Bronchoalveolar Lavage Fluids

At 29 days, the nasal lavage fluid (NALF) and bronchoalveolar lavage fluid (BALF) were collected. Briefly, the upper level of the trachea was ligated and a 22-gauge catheter was inserted into the nasopharynx. The nasal cavities were gently perfused with 1 ml cold PBS, and NALF was collected from the nose. BALF was obtained after lavage with 1 ml of cold PBS via a 20-gauge needle inserted through the upper part of the trachea. After centrifugation, the cells present within the NALF and BALF were counted using a hemocytometer and then cytospun onto glass slides and stained with Diff-Quick (Baso Diagnostics Inc., ZhuHai, Guangdong, People's Republic of China; http://www.baso-diagnostics.com). A total of 300 cells per slide were evaluated for eosinophils, macrophages, neutrophils, and lymphocytes at ×400 magnification, as previously reported [17]. The levels of IL-4, IL-5, IL-13, and interferon (IFN)-γ in the supernatants were measured using sandwich enzyme-linked immuno sorbent assay (ELISA) analysis following the manufacturer's instructions (R&D Systems, Minneapolis, MN, http://www.rndsystems.com).

### Lung and Nasal Histology and Inflammation Scoring

Lung and nasal tissues were removed after the lavage and fixed in 10% neutral formalin for 36 hours. The lungs were then embedded in paraffin, and nasal tissues were embedded in paraffin after being decalcified. Nasal tissues were prepared in a coronal plane at a distance of 5 mm from the nasal vestibule, and lung sections were prepared at a thickness of 4 μm. The sections were then stained with hematoxylin and eosin (H&E) for nasal tissues, and for lung tissues they were stained with both H&E and periodic acid–Schiff (PAS). The number of eosinophils was evaluated in the submucosal area of the whole nasal septum by microscopy (×400 magnification). Goblet cell (PAS positive cell) counts and inflammation score in the lungs were performed in a blinded fashion using a reproducible scoring system, as previously described [28, 29]. Briefly, for quantifying the lung inflammation, five sections across the main bronchus of each animal were randomly selected and given scores ranging from 0 to 3 based on the level of peribronchial inflammation and perivascular inflammation. The values were given according to the following inflammatory parameters: 0 when no inflammation was detectable; 1 for occasional cuffing with inflammatory cells; 2 for most bronchi or vessels surrounded by a thin layer (1–5 cells) of inflammatory cells; and 3 when most bronchi or vessels were surrounded by a thick layer (more than five cells) of inflammatory cells. For quantifying the goblet cell hyperplasia, the percentage of PAS-positive cells in epithelial areas was examined from 8 to 10 tissue sections per mouse.

### Measurement of Serum Immunoglobulin Response

The levels of OVA-specific IgE and IgG1 in serum were assayed using commercial ELISA kits (Shibayagi, Shibukawa, Gunma, Japan) following the manufacturer's instructions.

### Statistical Analysis

The experimental data were expressed as the mean ± SEM. Statistical analysis was performed using a one-way analysis of variance followed by a Student-Newman-Keuls test for multiple comparisons of the data with Gaussian distribution. A Kruskal–Wallis rank sum test followed by a Mann–Whitney *U* test was performed for two-group comparisons of the data with abnormal distribution. Statistical analysis was performed using GraphPad Prism 5 software (San Diego, CA), and *p* < .05 was considered statistically significant.

## RESULTS

### Systemic Administration of Human iPSC-MSCs Suppresses Allergic Nasal Symptoms

To determine whether human iPSC-MSCs have immunomodulatory functions in allergic inflammation, a mouse model of asthma and AR was developed. First, we investigated whether the administration of human iPSC-MSCs inhibited the occurrence of nasal symptoms. The frequency of sneezing and nasal rubbing in 10 minutes after the final challenge in the OVA/OVA/PBS group was significantly higher than that in the PBS group (*p* < .05) (Supporting Information Fig. 1A, 1B). However, treatment with iMR90-iPSC-MSCs (*p* < .05), N1-iPSC-MSCs (*p* < .01), and BM-MSCs (*p* < .05) before the challenge phase significantly reduced sneezing and nasal rubbing. The N1-iPSC-MSCs had a better inhibitory effect on sneezing when compared with the iMR90-iPSC-MSCs (*p* < .01). Interestingly, we found that the treatment with iPSC-MSCs before the sensitization phase had similar effects on reducing nasal rubbing and sneezing (*p* < .01). There was no difference in the nasal symptoms between naïve/naïve/iPSC-MSC mice and PBS-challenged mice. These data suggest that both human iPSC-MSCs and BM-MSCs have a comparable suppressive effect on allergic nasal symptoms.

### Systemic Administration of Human iPSC-MSCs Reduces OVA-Induced Upper and Lower Airway Inflammation

**Figure 2:**
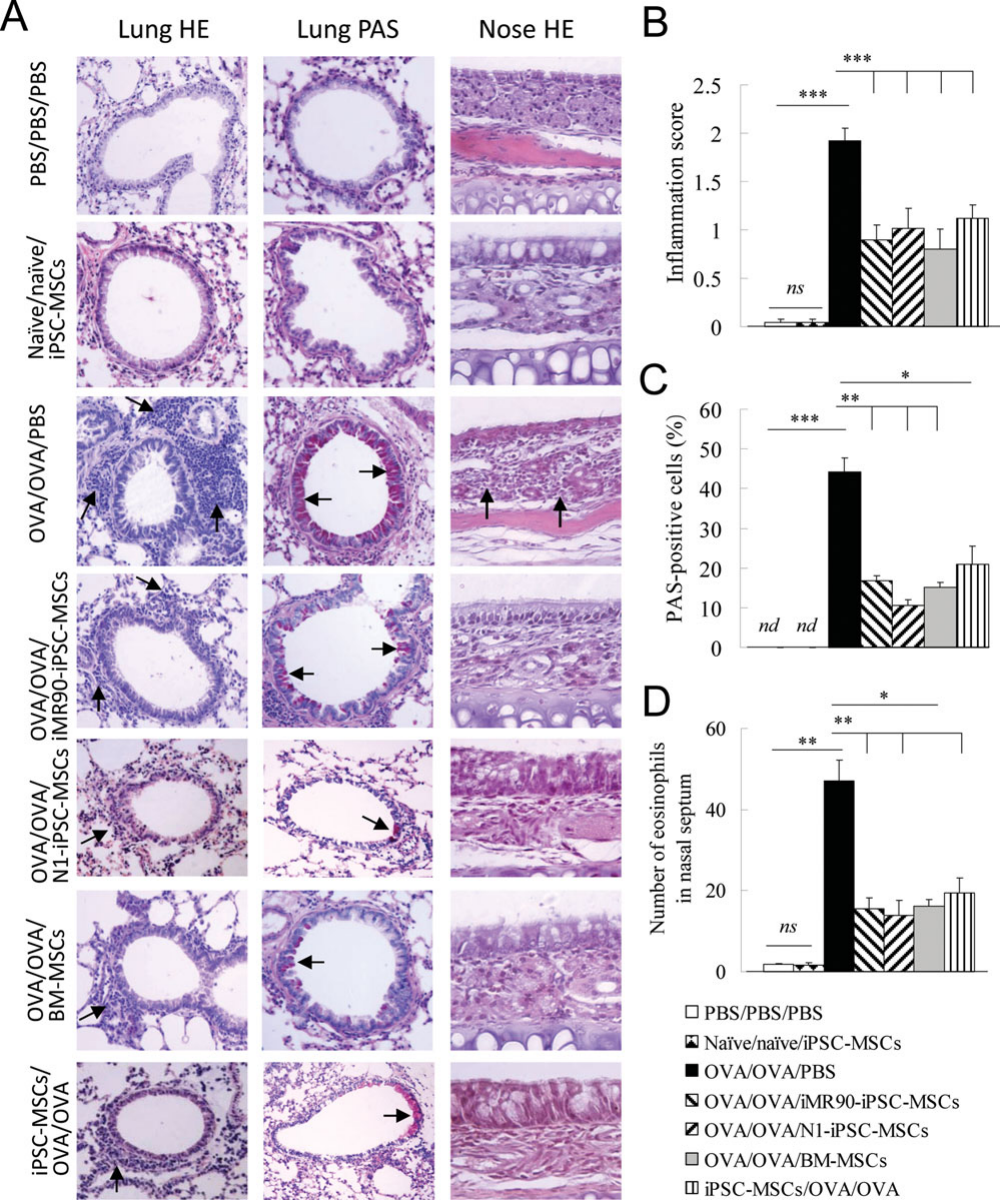
Administration of human iPSC-MSCs attenuated OVA-induced airway inflammation in the lung and nose. **(A):** Representative photomicrographs of H & E- and PAS-stained lung sections and H & E-stained nose sections from each group. Note the significant increase in inflammatory infiltrates (lung H&E, shown by arrows) and mucus accumulation at the luminal surface of the bronchi (lung PAS staining, shown by arrows) and eosinophil infiltrates in the nose (nose H&E, shown by arrows) in the OVA/OVA/PBS group. Original magnification ×200 (lung) and ×400 (nose). Statistical analysis for inflammation score **(B)** and mucus hypersecretion as quantified by PAS scores **(C)** in the lungs, and eosinophilic infiltration in the nasal submucosa **(D)**. Mean ± SEM. *, *p* < .05; **, *p* < .01; ***, *p* < .001 by Kruskal–Wallis rank sum test followed by the Mann–Whitney *U* test for two-group comparisons. Abbreviations: BM-MSC, bome marrow-derived mesenchymal stem cell; H&E, hematoxylin and eosin; iPSC-MSC, induced pluripotent stem cell; nd, not detectable; ns, no significance; OVA, ovalbumin; PAS, periodic acid–Schiff; PBS, phosphate-buffered saline.

We qualitatively and quantitatively evaluated the effect of human iPSC-MSC treatment before the challenge phase on the histopathology of the lung and nose. OVA/OVA/PBS mice exhibited obvious inflammatory cell infiltration (*p* < .001) in the peribronchial tissue, goblet cell hyperplasia as shown by PAS-positive cells in the bronchi (*p* < .001) and eosinophilic infiltration in the nasal submucosa (*p* < .01) (Fig. 2A–2D). Treatment with both clones of human iPSC-MSCs resulted in a significant decrease in lower airway inflammation (*p* < .001), goblet cell hyperplasia (*p* < .01), and upper airway inflammation (*p* < .01). Similar inhibitory effects were detected in the BM-MSC-treated mice (*p* < .001 for the inflammation score, *p* < .01 for the PAS-positive cells in the lungs, and *p* < .05 for eosinophils in the nasal septum) (Fig. 2A–2D). These data suggested that iPSC-MSCs had a comparable effect in attenuating allergic airway inflammation as human BM-MSCs. Moreover, we examined the effects of pretreating the mice with human iPSC-MSCs before the sensitization phase in our model. As illustrated in Figure 2, we observed a significant inhibition of inflammatory cell infiltration (*p* < .001) and goblet cell hyperplasia in the lungs (*p* < .05) as well as eosinophilic infiltration in the nose (*p* < .01). These data indicate that human iPSC-MSCs have the ability to inhibit the development of allergic inflammation during both the sensitization and challenge phases of the disease. In our study, no allergen-driven airway inflammation was found in naïve/naïve/iPSC-MSC mice. Although only a small number of transplanted iPSC-MSCs remained detectable in lung and nose tissues in iPSC-MSC-treated mice with OVA-induced allergic inflammation after transplantation, no transplanted iPSC-MSCs were detected in naïve/naïve/iPSC-MSC mice after transplantation (data not shown).

**Figure 3:**
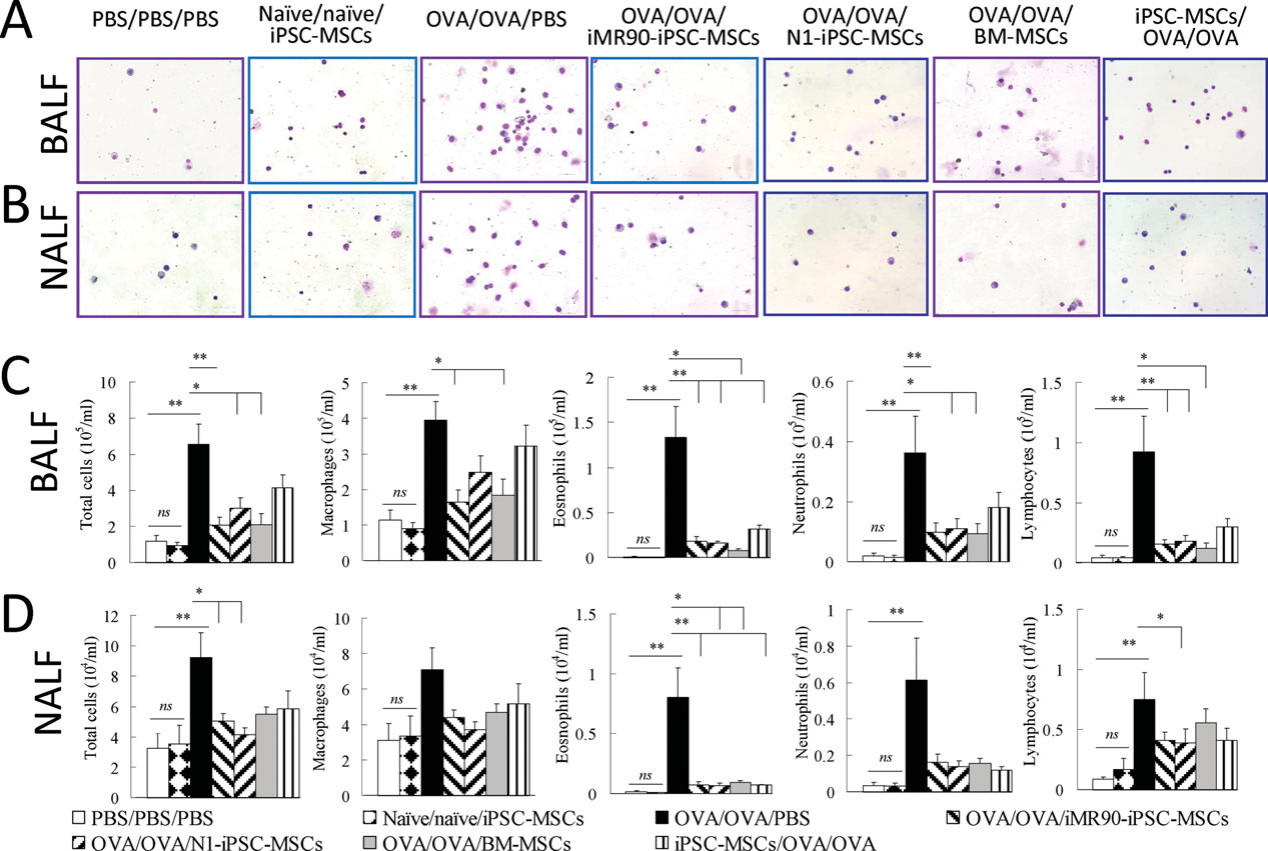
Systemic treatment with human iPSC-MSCs or BM-MSCs inhibits inflammatory cell infiltration in the BALF and NALF. Representative diff-quick staining of the inflammatory cells present in the BALF **(A)** and NALF **(B)**. Inflammatory cell counts in the BALF **(C)** and NALF **(D)**. Mean ± SEM. *, *p* < .05; **, *p* < .01 as conducted using a one-way analysis of variance and the Student-Newman-Keuls method for post hoc analysis for the total inflammatory cells, macrophages, and lymphocytes in the NALF, and using the Kruskal–Wallis rank sum test followed by the Mann–Whitney *U* test for two-group comparisons for the other parameters. Abbreviations: BALF, bronchoalveolar lavage fluid; BM-MSC, bone marrow-derived mesenchymal stem cell; iPSC-MSC, induced pluripotent stem cell-derived mesenchymal stem cell; NALF, nasal lavage fluid; ns, no significance; OVA, ovalbumin; PBS, phosphate-buffered saline.

We next examined the effect of human iPSC-MSCs on the OVA-induced inflammatory cell profiles in the BALF and NALF. The numbers of total and differential subsets of inflammatory cells increased in the BALF and in the NALF of OVA/OVA/PBS mice with the exception of macrophages (all *p* < .01, Fig. 3). However, treatment with both iMR90-iPSC-MSCs (*p* < .01) and N1-iPSC-MSCs (*p* < .05) 1 day before challenge phase significantly decreased the total number of inflammatory cells in the BALF. Differential staining demonstrated a decrease in eosinophils, macrophages, neutrophils, and lymphocytes upon two clone treatment but not macrophages upon treatment with N1-iPSC-MSCs (*p* < .05 or *p* < .01) (Fig. 3A, 3C). As for the NALF, similar effects were observed for total inflammatory cells and eosinophils upon treatment with both clones of iPSC-MSCs and for lymphocytes upon N1-iPSC-MSC treatment (*p* < .05 or *p* < .01, Fig. 3B, 3D). Treatment with BM-MSCs demonstrated the same effects on the total and the differential subsets of inflammatory cells in the BALF (all *p* < .05) and eosinophils in the NALF (*p* < .05). Interestingly, we also observed a significant decrease in eosinophils in both the BALF and NALF of animals that were pretreated with iPSC-MSCs before the sensitization phase (both *p* < .01). Treatment of naïve mice with human iPSC-MSCs alone did not affect the cell profiles of either the NALF or BALF compared with the PBS/PBS/PBS mice (*p* > .05). Taken together, our findings indicated that iPSC-MSCs were capable of attenuating the pathology observed in both the lung and nose in this mouse model of OVA-induced airway disease. Additionally, the iPSC-MSCs exerted their effect both on the sensitization phase and on the challenge phase.

### Systemic Administration of Human iPSC-MSCs Decreases Circulating Levels of OVA-Specific IgE

**Figure 4:**
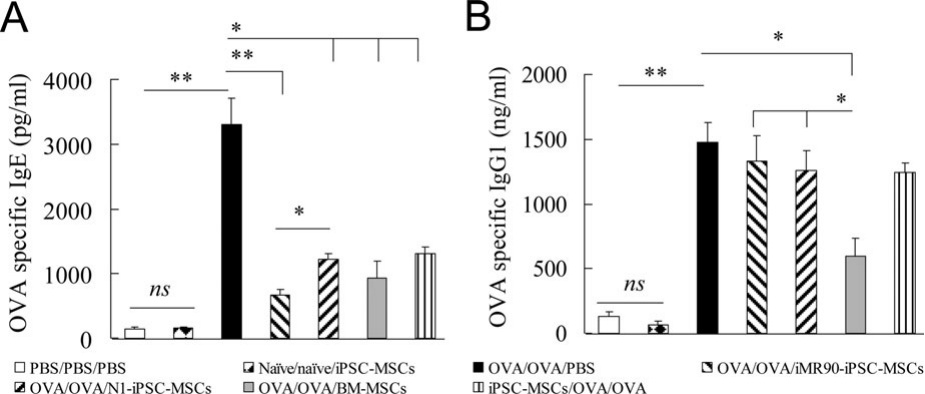
Systemic administration of human iPSC-MSCs or BM-MSCs decreases the serum levels of OVA-specific IgE. OVA-specific IgE **(A)** and IgG1 **(B)** levels in the sera of mice following the different treatments. The data are expressed as the mean ± SEM. *, *p* < .05; **, *p* < .01 by the Kruskal–Wallis rank sum test followed by the Mann–Whitney *U* test for two-group comparisons. Abbreviations: BM-MSC, bone marrow-derived mesenchymal stem cell; iPSC-MSC, induced pluripotent stem cell-derived mesenchymal stem cell; ns, no significance; OVA, ovalbumin; PBS, phosphate-buffered saline.

OVA sensitization and challenge is known to increase Th2-specific immunoglobulin concentrations in the sera. We confirmed a significant elevation in the serum levels of OVA-specific IgE and OVA-specific IgG1 (both *p* < .01) in our allergic model (Fig. 4). Systemic administration of both iMR90-iPSC-MSCs (*p* < .01) and N1-iPSC-MSCs (*p* < .05) 1 day before the challenge resulted in a significant decrease in serum OVA-specific IgE, but not IgG1 levels. Interestingly, we observed different levels of IgE between the iMR90-iPSC-MSC and N1-iPSC-MSC treatment (*p* < .05), suggesting that there is a different degree of allergic IgE inhibition by the different iPSC-MSC clones. Administration of BM-MSCs significantly decreased OVA-induced IgE and IgG1 serum levels (both *p* < .05), a result that was consistent with the decreased levels of specific IgE and IgG1 observed upon treatment with BM-MSCs in a mouse model of ragweed-induced asthma [18]. Moreover, we observed that the IgG1 levels were lower with the BM-MSC treatment when compared with both clones of iPSC-MSCs after OVA challenge (*p* < .05), suggesting their different abilities to regulate immunoglobulin production in allergic disease. Interestingly, the treatment of iPSC-MSCs before the sensitization significantly decreased OVA-specific IgE levels (*p* < .05). Treatment with human iPSC-MSCs in naïve mice did not affect the levels of either OVA-specific IgE or IgG1. Here, IgE represents the Th2 immune response, and it was significantly decreased by the treatments with the iPSC-MSC clones before sensitization and before challenge, indicating that the i.v. injection of iPSC-MSCs was responsible for downregulating the Th2 response in the allergic airway model.

### Systemic Administration of Human iPSC-MSCs Alters Cytokine Levels in Both the BALF and NALF

**Figure 5:**
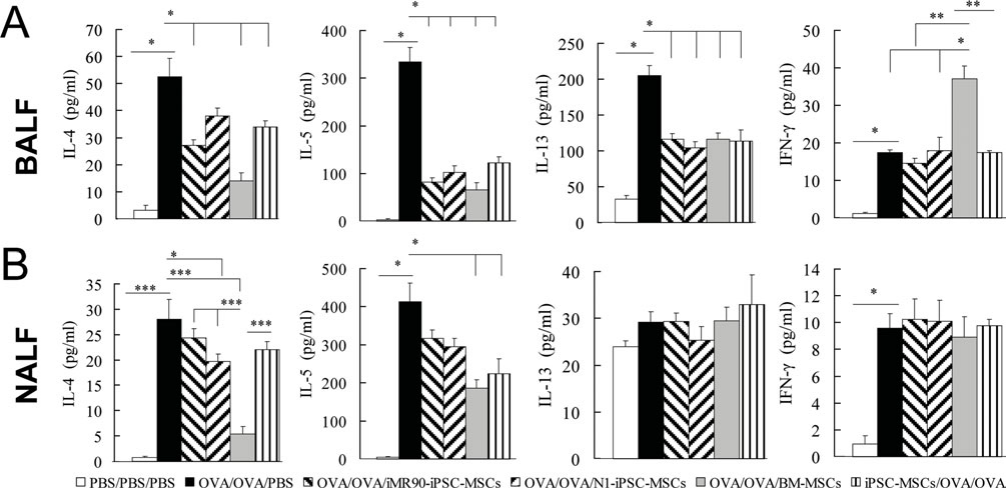
Systemic administration of human iPSC-MSCs inhibits the production of inflammatory cytokines in the BALF and NALF. The cytokine levels in the BALF and NALF were measured using enzyme-linked immunosorbent assay. The data are expressed as the mean ± SEM. *, *p* < .05; **, *p* < .01; ***, *p* < .001 as measured by a one-way analysis of variance and the Student-Newman-Keuls method for post hoc analysis IL-13 in the BALF and IL-4 in the NALF, and by the Kruskal–Wallis rank sum test followed by the Mann-Whitney *U* test for two-group comparisons for the other parameters. Abbreviations: BALF, bronchoalveolar lavage fluid; IFN, interferon; IL: interleukin; iPSC, induced pluripotent stem cell; MSC, mesenchymal stem cell; NALF: nasal lavage fluid; OVA, ovalbumin; PBS, phosphate-buffered saline.

We next determined the levels of the T helper cell-derived cytokines that were present in the BALF and NALF after administration of human iPSC-MSCs and BM-MSCs (Fig. 5). OVA-challenged mice showed increased levels of IL-4, IL-5, and IL-13 in the BALF (all *p* < .05), and IL-4 (*p* < .001) and IL-5 in the NALF (*p* < .05). These cytokines are characteristic of Th2 allergic inflammation. In contrast, treatment with human iMR90-iPSC-MSCs resulted in a significant decrease in IL-4, IL-5, and IL-13 (all *p* < .05) in the BALF, and treatment with N1-iPSC-MSCs resulted in a significant decrease in IL-5 and IL-13 in the BALF and IL-4 in the NALF (all *p* < .05). Treatment with BM-MSCs not only showed a similar effect in decreasing IL-4, IL-5, and IL-13 in the BALF (all *p* < .05) but also reduced the levels of IL-4 (*p* < .001) and IL-5 (*p* < .05) in the NALF. Furthermore, pretreatment with iPSC-MSCs before the sensitization phase significantly reduced the levels of IL-4, IL-5, and IL-13 in the BALF and IL-5 in the NALF (all *p* < .05). High levels of IFN-γ were observed in both the BALF and NALF in OVA/OVA/PBS mice (both *p* < .05, Fig. 5), and there were no differences after treatment with both clones of iPSC-MSCs. Treatment with BM-MSCs increased the levels of IFN-γ in the BALF when compared with OVA/OVA/PBS mice and three group mice treated with the iPSC-MSC clones (*p* < .05 or *p* < .01). These data suggest that iPSC-MSC treatment reduced the levels of Th2 cytokines in allergic inflammation, especially in the BALF. The differences between the effect of iPSC-MSCs and BM-MSCs on the cytokine levels in the NALF may be due to different MSC properties and different secretome profiles of MSCs derived from iPSCs or bone marrow.

## DISCUSSION

The immunomodulatory function of MSCs makes them promising candidates for allergic disease therapy. MSCs generated from iPSCs are morphologically and functionally similar to BM-MSCs [10]. Because of the limited proliferative capacity and the rapid loss of differentiation potential of adult MSCs [5–7], iPSC-MSCs with an unlimited resource might provide an alternative source of stem cells for therapeutic use.

In this study, we induced allergic inflammation in both the upper and lower airways of mice. Similar to BM-MSCs, we demonstrated that human iPSC-MSCs significantly inhibited nasal eosinophilia and lung pathology, decreased the levels of Th2 cytokines in the NALF and BALF and decreased IgE levels in the serum. These data are consistent with previous studies that MSCs from BM or adipose tissues attenuated allergic responses in asthma and AR [18–21, 30, 31]. More importantly, the above findings were confirmed using two different clones of human iPSC-MSCs, indicating their stable capability to regulate the immune response during allergic inflammation. Some small differences were found in the effect between the two iPSC-MSCs clones, such as macrophage numbers and IL-4 levels in the BALF and lymphocyte numbers in the NALF. These differences may be due to differences in the cell sources of iPSCs, the process of induction, the culture conditions, and even the passages. Considering that the immunosuppressive function of iPSC-MSCs is maintained during multiple passages without any senescence in contrast to BM-MSCs [2, 11], it is plausible that iPSC-MSCs may offer a long-lasting, more efficient immunoregulatory function to allergic disease than BM-MSCs. To our knowledge, this is the first study to investigate the potential role of iPSC-MSCs in the modulation of the allergic response in an animal model.

Interestingly, we found that pretreatment with iPSC-MSCs before the sensitization phase exhibited similar, if sometimes weaker, effects on allergic inflammation when compared with iPSC-MSC administration before the challenge phase. These data are consistent with a previous study in which treatment with human gingiva-derived MSCs before both the sensitization and challenge phases attenuated contact hypersensitivity [32]. In the development of allergic disorders, DC and allergy-specific T cells are involved in the sensitization phase, and allergy-specific T cells and mast cells are involved in the challenge phase [16]. BM-MSCs were demonstrated to affect the functions of DCs [33], T cells [34], and mast cells [35]. Similarly, we have found that human iPSC-MSCs inhibit the proliferation of T lymphocytes and modulate T-cell phenotypes in AR [23]. Our findings indicate that iPSC-MSCs may work on different cells in different phases of allergic inflammation, suggesting a broad immunomodulation by iPSC-MSCs. It allows the iPSC-MSCs to rectify the imbalance of the immune responses in different environments.

We found some differences in the effect of iPSC-MSCs and BM-MSCs on the serum levels of IgG1, the levels of IL-4 and IL-5 in the NALF, and the IFN-γ levels in the BALF. Similar to our study, BM-MSCs were demonstrated to increase IFN-γ levels [17, 21] and decrease IgG1 levels [18] in an asthma model. These different effects may be related to the different properties and different secretome profiles of MSCs derived from adult tissues or pluripotent stem cells. iPSC-MSCs displayed different proliferative capacity and telomerase activity when compared with BM-MSCs [10]. Furthermore, using a cytokine antibody array, we found that conditioned medium from human embryonic stem cell (ESC)-MSCs and BM-MSCs had many common characteristics but also some distinct differences in paracrine factor profiling, such as in human originated angiogenin [36]. We also demonstrated a difference in the secretome profiles between iPSC-MSCs and BM-MSCs against anthracclines-induced cardiomyopathy (Q. Lian et al., manuscript submitted for publication). The differential paracrine factor profile between iPSC-MSCs and BM-MSCs might account for some of the differences between their effects on the levels of cytokines and IgG1 in response to OVA-induced allergy.

There are some limitations of this study. An important limitation is that the study of immune rejection after human iPSC-MSCs were transplanted into immunocompetent mice is lacking. Nevertheless, studies of human MSCs from adult tissues in immunocompetent animals without using immunosuppressants have been performed, and many studies have demonstrated that human MSCs have therapeutic potential in immunocompetent rodent models of immune diseases, such as asthma [19, 20], AR [22], contact hypersensitivity [32], and systemic lupus erythematosus [37, 38]. Similarly, we demonstrated the therapeutic activity of human iPSC-MSCs to allergic airway inflammation in immunocompetent mice. We acknowledge the possibility that immune rejection could occur in our study. It should be further studied in immnuocompromised mice in the future. In addition, it should nonetheless be noted that the immunomodulatory properties of human iPSC-MSCs in the attenuation of allergic inflammation would be desirable, there are challenges to overcome prior to their clinical application. Although we and other labs have not observed the teratogenic effects of human ESC/iPSC-MSCs in animal studies [36, 39], the safety concerns on genomic instability of iPSCs and ESCs need to be evaluated carefully before clinical translation [40, 41].

## CONCLUSION

In summary, we demonstrated that similar to BM-MSCs, the administration of iPSC-MSCs significantly suppressed the pathology and permitted a re-balance in the immune response in a mouse model of allergic inflammation in both the upper and lower airways. Pluripotent stem cells potentially offer an unlimited source for functional and mass MSC generation. Our data provide preliminary evidence that iPSC-MSCs may be used as a novel alternative to adult MSCs in the treatment of allergic airway diseases.

## Supplementary material

Supplementary Figure 1. Systemic administration of human iPSC-MSCs or BM-MSCs suppressed nasal symptoms. (A) Sneezing score. (B) Nasal rubbing score. *P < 0.05, ** P < 0.01 by the Mann–Whitney U test for two-group comparisons. ns: no significance.
